# Lazarillo-related Lipocalins confer long-term protection against type I Spinocerebellar Ataxia degeneration contributing to optimize selective autophagy

**DOI:** 10.1186/s13024-015-0009-8

**Published:** 2015-03-19

**Authors:** Manuela del Caño-Espinel, Judith R Acebes, Diego Sanchez, Maria D Ganfornina

**Affiliations:** Instituto de Biología y Genética Molecular-Departamento de Bioquímica y Biología Molecular y Fisiología, Universidad de Valladolid-CSIC, c/ Sanz y Forés 3, 47003 Valladolid, Spain

**Keywords:** Autophagy, Neurodegeneration, Neuroprotection, Polyubiquitinated protein clearance, Drosophila, GLaz, NLaz, ApoD, GstS1, Atg1/ULK1, Atg4a, Atg8a/LC3, p62

## Abstract

**Background:**

A diverse set of neurodegenerative disorders are caused by abnormal extensions of polyglutamine (poly-Q) stretches in various, functionally unrelated proteins. A common feature of these diseases is altered proteostasis. Autophagy induction is part of the endogenous response to poly-Q protein expression. However, if autophagy is not resolved properly, clearance of toxic proteins or aggregates cannot occur effectively. Likewise, excessive autophagy induction can cause autophagic stress and neurodegeneration. The Lipocalins ApoD, Glial Lazarillo (GLaz) and Neural Lazarillo (NLaz) are neuroprotectors upon oxidative stress or aging. In this work we test whether these Lipocalins also protect against poly-Q-triggered deterioration of protein quality control systems.

**Results:**

Using a Drosophila retinal degeneration model of Type-1 Spinocerebellar Ataxia (SCA1) combined with genetic manipulation of NLaz and GLaz expression, we demonstrate that both Lipocalins protect against SCA1 neurodegeneration. They are part of the endogenous transcriptional response to SCA1, and their effect is non-additive, suggesting participation in a similar mechanism. GLaz beneficial effects persist throughout aging, and appears when expressed by degenerating neurons or by retinal support and glial cells. GLaz gain-of-function reduces cell death and the extent of ubiquitinated proteins accumulation, and decreases the expression of Atg8a/LC3, p62 mRNA and protein levels, and GstS1 induction. Over-expression of GLaz is able to reduce p62 and ubiquitinated proteins levels when rapamycin-dependent and SCA1-dependent inductions of autophagy are combined. In the absence of neurodegeneration, GLaz loss-of-function increases Atg8a/LC3 mRNA and p62 protein levels without altering p62 mRNA levels. Knocking-down autophagy, by interfering with Atg8a or p62 expression or by expressing dominant-negative Atg1/ULK1 or Atg4a transgenes, rescues SCA1-dependent neurodegeneration in a similar extent to the protective effect of GLaz. Further GLaz-dependent improvement is concealed.

**Conclusions:**

This work shows for the first time that a Lipocalin rescues neurons from pathogenic SCA1 degeneration by optimizing clearance of aggregation-prone proteins. GLaz modulates key autophagy genes and lipid-peroxide clearance responsive genes. Down-regulation of selective autophagy causes similar and non-additive rescuing effects. These data suggest that SCA1 neurodegeneration concurs with autophagic stress, and places Lazarillo-related Lipocalins as valuable players in the endogenous protection against the two major contributors to aging and neurodegeneration: ROS-dependent damage and proteostasis deterioration.

**Electronic supplementary material:**

The online version of this article (doi:10.1186/s13024-015-0009-8) contains supplementary material, which is available to authorized users.

## Background

A diverse set of neurodegenerative disorders are caused by abnormal extensions of polyglutamine (poly-Q) stretches in various, functionally unrelated proteins. No effective treatment to prevent or slow down the progression of poly-Q-based neurodegeneration is currently available. Since anomalous glutamine extensions lead to protein misfolding and aggregation, independently of the particular protein affected, all of these diseases display altered cellular proteostasis [1] as a common feature. The mechanisms engaged in protein quality control, mainly the ubiquitin-proteasome and autophagy systems, are obvious candidate processes on which to focus therapeutic manipulations, aiming at the clearance of mutant proteins. Because poly-Q proteins tend to oligomerize, autophagy is thought to play a prominent role in their management. However, autophagic responses to pathological damage are not yet well understood. Very diverse modes of experimental or pharmacological induction of autophagy have proven to be protective in poly-Q disease models (reviewed in [2]). Also, induction of autophagy has been proposed to be part of the natural response of cells upon glutamine-extended protein expression [3]. However, increased levels of autophagy do not always have beneficial effects on poly-Q-based neurodegeneration. Nisoli et al. [4] showed that induction of autophagy does not rescue the neurodegeneration caused by poly-Q-extended atrophin-1 in a fly model of DRPLA (dentatorubral-pallidoluysian atrophy). If autophagy is induced but not resolved properly, clearance of damaged and unfolded proteins cannot take place effectively. Also, excessive or imbalanced induction of autophagy might be deleterious if autophagosome turnover is unable to keep pace with its formation [5]. This situation will produce autophagic stress, which can actively contribute to neuronal atrophy, neurite degeneration and cell death [6,7].

We have studied the functions of an evolutionarily-related group of proteins belonging to the Lipocalin family, named Lazarillo in invertebrates (Laz, GLaz and NLaz) and Apolipoprotein D (ApoD) in vertebrates. They are secreted proteins with a single globular ß-barrel domain that forms a “cup-like” structure with ability to bind small molecules, mostly hydrophobic in nature [8]. Hydrophobic ligands as varied as retinoic acid, arachidonic acid, progesterone, sphingomyelin, the pheromone 7(d)tricosene, or the endocannabinoid anandamide, have been shown to bind to some of these Lazarillo-related Lipocalins [9-11].

Lazarillo-related Lipocalins show neuroprotective effects in mice and lifespan extending abilities in flies, both under normal conditions and upon exposure to oxidative stress or injury [12-21]. These neuroprotective functions are mediated either by counteracting lipid peroxidation or by controlling myelin phagocytosis in injured nerves [20,22]. Moreover, ApoD stands among a small set of genes whose over-expression upon nervous system aging is conserved in mammals [23,24], and its over-expression is extensively correlated with a wide spectrum of nervous system damage, like stroke or meningoencephalitis, psychiatric disorders like schizophrenia or bipolar disorder, and neurodegenerative diseases like Alzheimer, Parkinson, Huntington, Niemann Pick or multiple sclerosis (reviewed in [25-27]).

Physiological aging and age-related pathologies share many causal factors (reviewed in [28]), and it is not surprising that protective agents are also shared, even though the particular form or the rate of damage accumulation might vary. Two of the major factors contributing to aging and neurodegeneration are (1) reactive oxygen species (ROS)-mediated damage and (2) deterioration of protein and organelle quality control systems.

We have previously tested the protective effect of a Lazarillo-related Lipocalin, GLaz, on the degeneration caused by a primarily mitochondrial dysfunction disease, Friedreich ataxia, a neurodegenerative disorder with a redox imbalance as a major contributor to its pathophysiology. GLaz is able to counteract some of the effects of frataxin deficiency, reducing the level of lipid peroxidation and free fatty acids, and leading to improved survival of frataxin-deficient flies [29]. Yet, given the variety of neurodegenerative conditions that concur with ApoD over-expression, these Lipocalins are probably important elements of the shared set of protective agents. Thus, it is pertinent to ask whether Lipocalins can protect from neurodegenerative disorders with very different etiology, such as the poly-Q based pathologies.

We therefore set to study the effects of the Drosophila Lipocalins GLaz and NLaz on a poly-Q triggered ataxia model, the Type I Spinocerebellar Ataxia (SCA1) [30], to focus on their effect on protein and organelle quality control systems, the other major factor whose deterioration is causative to aging. Until now, no Lipocalin has been tested for its ability to modify poly-Q-based neurodegeneration and this work represents a first test for extracellular lipid-binding proteins as modulators of proteostasis under neurodegenerative conditions.

## Results

### The Lipocalin GLaz is up-regulated upon neurodegeneration by pathogenic human Ataxin 1, and it is expressed locally in the fly retina

The expression of human ApoD is boosted upon various neurodegenerative conditions (for reviews see [25-27]), but SCA1 is still unexplored. We therefore tested whether the Drosophila Lipocalins change their expression in SCA1-affected flies.

**Figure 1:**
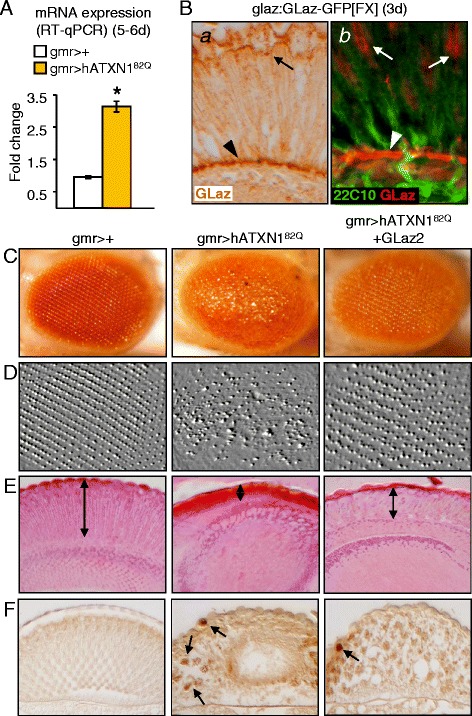
**GLaz expression and rescue with the GAL4/UAS system of the SCA1 neurodegeneration model in Drosophila. A**, RT-qPCR levels of GLaz transcripts in 5–6 day old fly heads. Statistical differences assayed by Mann–Whitney U-test. *p < 0.05. **B**, Native over-expression of GLaz using the direct reporter transgene glaz:GLaz-GFP[FX]. Arrows point to GLaz-positive retinal support cells, and arrowhead indicates GLaz-positive basal glial cells. **C-F**, Adult (5-6d old) Drosophila eye morphology of control (gmr > +), degenerated (gmr > hATXN1^82Q^) and rescue test (gmr > hATXN1^82Q^ + GLaz2). Representative examples of light microscopy **(C)**, surface filter-processed image of the central retinal region in C **(D)**, hematoxilin/eosin histochemistry of paraffin sections **(E)**, and Ataxin 1 immunohistochemistry on paraffin sections **(F)** are shown. Double arrows mark the retina thickness in the histological sections **(E)**, and arrows point to inclusion bodies of the poly-Q protein **(F)**.

To assay expression of the Drosophila Lipocalins in a poly-Q-based neurodegenerative disease, we have used the GAL4/UAS system to express a pathogenic version of human Ataxin 1 with an expanded glutamine tract (hATXN1^82Q^) in Drosophila retinal photoreceptors using the gmr:GAL4 driver. In this model of SCA1 [30] photoreceptors accumulate nuclear inclusions of the human protein and start degenerating during late pupal stage when flies develop at 25°C. We first tested GLaz expression. Figure 1A shows that its expression increases 3-fold in the photoreceptors degeneration model of SCA1 when assaying mRNA levels in 5–6 days old fly heads.

Since these results demonstrate that GLaz, like ApoD in vertebrates, is part of the natural response of the tissue to the neurodegenerative insult, we characterized its expression in the Drosophila eye, searching for cells that might influence the tissue response to the neurodegenerative condition. To assay for GLaz expression we used two independent insertions of a fusion protein construct (glaz:GLaz-GFP[FX] and glaz:GLaz-GFP[F2] [12]). These reporters show GFP-positive cells in the retina (Figure 1B). The GLaz signal does not co-localize with the neuronal marker 22C10 (Figure 1Bb), suggesting that support retinal cells (arrows in Figure 1B) and retinal basal glia (arrowhead in Figure 1B) are the source of this Lipocalin.

### Co-expression of GLaz with extended poly-Q human Ataxin 1 ameliorates photoreceptor degeneration

To test whether GLaz is able to protect cells that are degenerating due to the expression of hATXN1^82Q^, we either expressed the Lipocalin in the degenerating photoreceptors (using the same controller to drive the expression of the poly-Q protein and GLaz), or in the cells naturally expressing the GLaz protein under the control of its native promoter (using a glaz:GLaz-GFP construct).

We first demonstrated that the expression of the UAS:GLaz transgene does not directly cause an alteration in the amount of hATXN1^82Q^ produced in photoreceptors by quantifying the hATXN1^82Q^ immunolabeling signal in eye imaginal discs of 3^rd^ instar larva (Additional file 1). This time point was chosen to avoid variation in the number of of hATXN1^82Q^-expressing cells, since cell death caused by neurodegeneration is not present yet [30].

**Figure 2:**
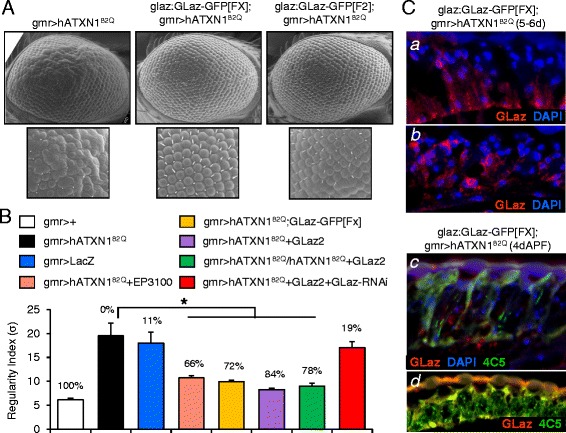
**Rescue of hATXN1** ^**82Q**^ **-dependent photoreceptor degeneration by GLaz over-expressed with a native spatiotemporal pattern. A**, Representative examples of scanning electron microscopy and magnified images of retinas degenerated (gmr > hATXN1^82Q^), and co-expressing different GLaz expression transgenes (glaz:GLaz-GFP[FX] and glaz:GLaz-GFP[F2]). **B**, Quantitative estimate of degeneration by computing a regularity index based on the variance (σ) of the local intensity maxima distances. A percent recovery of the degeneration is indicated for each genotype. 20–35 eyes/genotype were used to compute regularity indexes. Average ± S.E.M. are represented. Asterisk represents statistically significant differences (Student’s t-test, *P < 0.05) with respect to the degenerated gmr > hATXN1^82Q^ genotype. **C**, **a**,**b**: GLaz expression revealed by GFP fluorescence immunohistochemistry of paraffin sections of retinas of flies expressing hATXN1^82Q^ and the transgene glaz:GLaz-GFP[FX]. Nuclei are shown with DAPI staining. **c**,**d**: Double fluorescence immunohistochemistry revealing GLaz and the photoreceptor marker rhodopsin (4C5). DAPI stains nuclei in C.

GLaz produces a significant rescue of photoreceptor degeneration when co-expressed with hATXN1^82Q^ (Figure 1C-F, Additional file 2). Two UAS:GLaz and two EP insertions upstream of GLaz were used to drive the expression of the Lipocalin under the control of gmr:GAL4. The adult eye external morphology was inspected (Figure 1C; Additional file 2A) and the degree of surface regularity analyzed (Figure 1D) and quantified (Figure 2B) by estimating a regularity index according to the variance of intensity maxima (See Methods section; [31]). Histochemistry of paraffin sections (Figure 1E) shows severe atrophy of retinal cells in the hATXN1^82Q^-expressing flies, while the retinal epithelial pattern is reasonably preserved in the presence of gain-of-function constructs of GLaz. The degeneration observed in hATXN1^82Q^-expressing photoreceptors of 3 days old flies is already extensive, and no further deleterious effect is observed when native GLaz expression is eliminated in the null mutant background (Additional file 3A). The expression of an unrelated control protein (LacZ) does not rescue the degeneration phenotype of the SCA1 model (Additional file 2C, Figure 2B). Specificity was further tested by simultaneously over-expressing GLaz in photoreceptors (with the UAS:GLaz2) and decreasing the expression of GLaz with a UAS:GLazRNAi construct. The rescue is abolished in this situation (Additional file 3B, Figure 2B). Moreover, the rescue observed with GLaz gain-of-function is dependent on temperature (due to the temperature sensitivity of the GAL4/UAS system) and the transgene expression levels. The GLaz-triggered rescue at 25°C shows a similar extent when comparing flies with either one or two copies of the UAS:hATXN1^82Q^ transgene located in the 1^st^ chromosome (Additional file 2D, Figure 2B). However, a lesser rescue was obtained either at 29°C or with a 3^rd^ chromosome UAS:hATXN1^82Q^ insertion line that shows a higher expression level (results not shown).

Human Ataxin 1 was detected by immunohistochemistry in the retina sections (Figure 1F). Nuclear inclusion bodies of the extended poly-Q protein are evident in the photoreceptors expressing hATXN1^82Q^ as previously described [30]. The expression of GLaz does not fully abolish the formation of these protein aggregates (arrows in Figure 1F) in spite of the preserved tissue integrity.

Taken together our data show that GLaz ameliorates the hATXN1^82Q^-provoked degeneration, but there seems to be a limited range of the disease severity where GLaz can exert a beneficial effect when expressed by the degenerating photoreceptors.

### GLaz rescues degeneration of photoreceptor neurons in a stable manner when produced by its native cell expression domain

Since the Lazarillo-related fly Lipocalins are secreted proteins [9,32], we further tested if GLaz expressed with its native spatiotemporal pattern would be able to protect photoreceptors when accessing the degenerating cell from the extracellular space. We tested the effect of GLaz gain-of-function by expressing, in wild type background, the GLaz-GFP fusion protein under the control of the native GLaz promoter (glaz:GLaz-GFP [F2] or glaz:GLaz-GFP[FX] insertion lines) on the pathogenic human poly-Q Ataxin 1-generated photoreceptor degeneration (Figure 2A,B and Additional file 4). The rescue is present from early stages of degeneration in the pupal eyes, 5 days after pupa formation (APF), to aged adult flies (30 days old) (Additional file 4). These results show that the rescue does not require Lipocalin expression in the degenerating cell, and that the GLaz protecting effect can be exerted when the source of protein is a different cell. The support and basal glial cells in the retina are the closest natural source of GLaz in this case (Figure 1B). Therefore GLaz can act as an extrinsic factor protecting the photoreceptors in a non-cell-autonomous manner. This paracrine neuroprotective mechanism is stable enough to maintain retinal rescue in aged flies (30 days old flies, Figure 2A and Additional file 4).

GLaz-GFP protein localization is evident in cells undergoing degeneration in 5–6 days old flies (Figure 2Ca,b). When analyzing mildly degenerated regions of retinas at 4d-APF, no GLaz-GFP signal is detected in the hATXN1^82Q^-expressing photoreceptors (Figure 2Cc). However, more degenerated regions of retina showed GLaz protein signal co-localizing with the photoreceptor marker 4C5 (Figure 2Cd). These results suggest a potential incorporation of the extracellular protein into the degenerating photoreceptors that resembles the internalization of exogenous ApoD observed in cultured astrocytes [18].

### The Lipocalin NLaz is up-regulated by pathogenic poly-Q Ataxin 1 expressed in the fly retina, and rescues photoreceptor neurodegeneration

**Figure 3:**
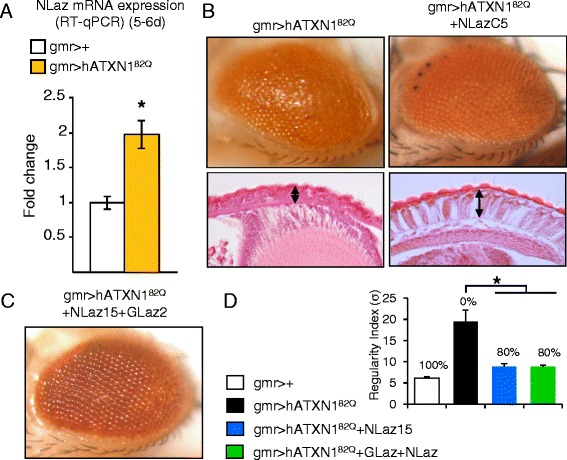
**NLaz is over-expressed in response to hATXN1** ^**82Q**^ **-dependent photoreceptor degeneration, and its over-expression with the GAL4/UAS system rescues the pathogenic phenotype. A**, RT-qPCR levels of NLaz mRNA in 5–6 day old fly heads. Statistical differences were assayed by Mann–Whitney U-test. *P < 0.05. **B**, Representative fly eyes and hematoxilin/eosin histochemistry of paraffin sections showing the degenerated phenotype (gmr > hATXN1^82Q^) and the NLaz rescue (gmr > hATXN1^82Q^ + NLazC5). Arrows mark the retina thickness in the histological sections. **C**, Photoreceptor degeneration rescue observed when co-expressing hATXN1^82Q^ with the Lipocalins NLaz and GLaz. **D**, Quantification of photoreceptor degeneration rescue for NLaz and the co-expression of NLaz and GLaz. Asterisk represents statistically significant differences (Student’s t-test, *P < 0.05) with respect to the degenerated gmr > hATXN1^82Q^ genotype.

The Drosophila Lipocalin NLaz is expressed by neurons in the fly CNS and PNS [32]. The native expression of NLaz increases 2-fold in retinal SCA1 model fly heads (Figure 3A). However, when NLaz expression was assayed by either a direct reporter construct (nlaz:GFP[R2]) or by a nlaz:GAL4 construct driving the expression of GFP (UAS:CD8-GFP), none of the NLaz reporters showed expression in the fly retina (not shown).

Nevertheless, co-expressing NLaz with hATXN1^82Q^ in retinal photoreceptors under the control of the gmr:GAL4 driver protects cells from degeneration (Figure 3B and Additional file 2B). The rescue level obtained with NLaz was similar to that with GLaz, as quantified by the degree of surface regularity (Figures 2B and 3D, 66-84% rescue with GLaz, 80% rescue with NLaz). Interestingly, the rescue attained by each Lipocalin is comparable to that obtained when both GLaz and NLaz are over-expressed in photoreceptors (Figure 3C,D, 80% rescue), suggesting that both proteins could be acting through the same mechanism and no additive effect is obtained.

Since GLaz is natively expressed in cells within the niche of the degenerating photoreceptors in the SCA1 model, we focused on this Lipocalin for the subsequent search of the cellular mechanism causative of neuroprotection.

### Over-expressing GLaz decreases apoptotic cell death in the presence of expanded human Ataxin 1

**Figure 4:**
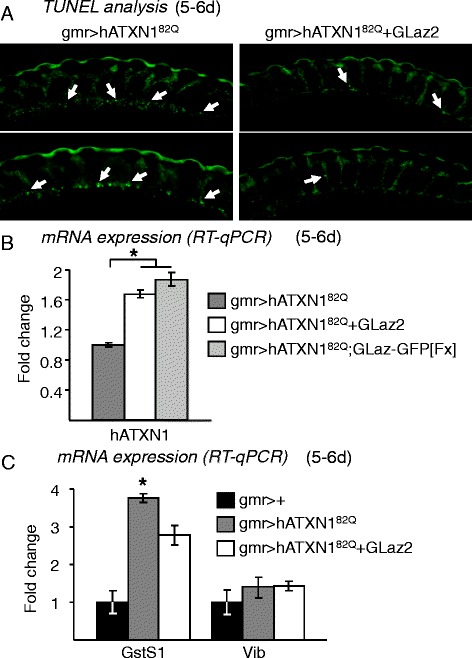
**Effects of GLaz over-expression on cell death due to hATXN1** ^**82Q**^ **-dependent degeneration, and on SCA1-modifiers expression. A**, TUNEL analysis of apoptotic cell death in paraffin sections of degenerated (gmr > hATXN1^82Q^) and GLaz rescued (gmr > hATXN1^82Q^ + GLaz2) retinas. Representative examples of retina sections showing a decreased number of apoptotic nuclei (white arrows) in flies that over-express GLaz. **B**, hATXN1 mRNA levels in fly heads of the SCA1 model and lines expressing a double dose of GLaz. **C**, RT-qPCR expression levels of the SCA1 neurodegeneration modifiers GstS1 and Vib. Statistical differences of RT-qPCR data were assayed by Mann–Whitney U-test. *P < 0.05.

Terminal deoxynucleotidyl transferase dUTP nick-end labeling (TUNEL) analysis reveals that photoreceptor cells die by apoptosis upon hATXN1^82Q^ expression (Figure 4A). GLaz over-expression causes a reduction in the number of apoptotic cells detected in the fly retina (Figure 4A). To have a quantitative estimate of the cell death rescue, we measured the levels of hATXN1 mRNA by qRT-PCR under two assumptions: (i) at pupal stage, hATXN1 mRNA levels are similar when degeneration is not yet present (hATXN1 protein levels show no difference, Additional file 1), and (ii) only intact photoreceptors would contribute significantly to the pool of detectable mRNA transcribed from the human transgene once the degeneration has started. GLaz does increase the levels of human Ataxin 1, both when co-expressed in photoreceptor neurons and when expressed by the nearby support cells and basal glial retinal cells (Figure 4B), in agreement with the decrease observed in the number of TUNEL-positive cells.

### GLaz alters the expression of a SCA1 phenotype-modifier controlling lipid peroxide clearance

Among the genes found to modify SCA1-induced neurodegeneration in Drosophila, two genes involved in lipid management were uncovered [30,33]: Glutathione S transferase S1 (GstS1), a homologue of the mammalian hematopoietic prostaglandin D synthase (HPGDS), and Vibrator (Vib) a phosphatidylinositol transfer protein, homologue of PITPN-alpha in vertebrates. Over-expression of GstS1 or Vib suppresses the neurodegeneration caused by the poly-Q human Ataxin 1, and the loss of function of GstS1 enhances the phenotype [30,33].

Although GstS1 is classified as part of the constitutively expressed oxidative stress defensive enzymes [34], no transcriptional regulation upon neurodegeneration has been described for GstS1 or Vib. We explored the putative transcriptional regulation of these two genes in our system. While no modification of Vib transcript levels was seen in the SCA1 model either alone or combined with over-expression of GLaz, a 4-fold induction of the GstS1 transcript was obtained upon hATXN1^82Q^-induced photoreceptor degeneration (Figure 4C). This induction was significantly reduced when GLaz is over-expressed (Figure 4C).

The direct cause of GstS1 up-regulation under SCA1-induced neurodegeneration remains to be explored. However, since GLaz controls lipid peroxide levels [12], we propose a mechanistic link between the lower levels of oxidized substrates upon GLaz gain-of-function and the decreased GstS1 transcription. Thus, the ectopic expression of poly-Q hAtaxin 1 results in the induction of both GLaz and GstS1 genes, and they contribute to an efficient lipid peroxide clearance. Other lipid management genes, like Vib, are not induced even though Vib is also a modifier of SCA1 neurodegenerative phenotype.

### SCA1-dependent build-up of ubiquitinated proteins is reduced by GLaz over-expression

**Figure 5:**
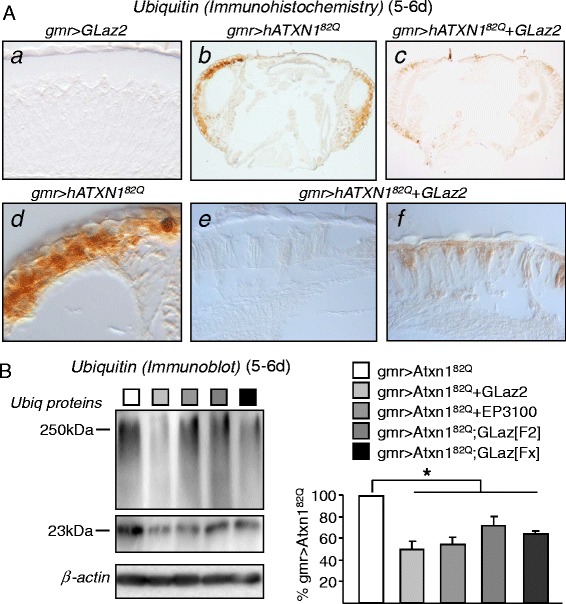
**Decrease of ubiquitinated proteins build-up attained by GLaz over-expression. A**, Ubiquitin HRP-immunohistochemistry on paraffin sections of control, degenerated and GLaz-rescued retinas shows the decrease in ubiquitin labeling when over-expressing GLaz. **B**, Ubiquitinated protein load measured by ubiquitin immunoblot signal in different lines expressing the pathogenic hATXN1^82Q^ alone or in combination with GLaz-expressing transgenes. Statistical differences were assayed by Student’s t-test. *P < 0.05.

To address whether GLaz over-expression modifies any of the protein quality control systems altered in the SCA1fly model, we analyzed by immunohistochemistry and immunoblot the expression pattern of ubiquitinated proteins (Figure 5). Abundant ubiquitinated protein aggregates are evident upon neurodegeneration (Figure 5Ab,d) when compared to controls (driver alone, not shown, or GLaz over-expression in wild type background, Figure 5Aa). This accumulation is greatly reduced when GLaz is co-expressed with hATXN1^82Q^ (Figure 5Ac,e,f). A quantitative analysis by immunoblot demonstrates that over-expressing GLaz, either in the degenerating photoreceptor or in the native GLaz expression domain, results in a significant decrease of ubiquitinated protein levels (Figure 5B).

### Over-expression of GLaz reduces SCA1-dependent Atg8a induction

A defense mechanism against lipid peroxides in a ROS-promoting environment, and a mechanism controlling proteostasis seem unconnected processes at first glance. However, links between these mechanisms are starting to emerge. GstS1 is oxidized while catalyzing the conjugation of lipophilic substrates with reduced glutathione, and this fact has another consequence: the induction of autophagy by the Jun-N-terminal Kinase (JNK) when this kinase is released from the inhibitory interaction of the reduced form of GstS1 protein [34]. On the other hand, ubiquitinated proteins are known to be degraded by autophagy in neurons [35-37]. Thus, we hypothesized that the protective effects of proteins like Lipocalins, influencing both lipid oxidation and proteostasis, might be related to the membrane-mediated process of autophagy, potentially adding another link between these processes.

**Figure 6:**
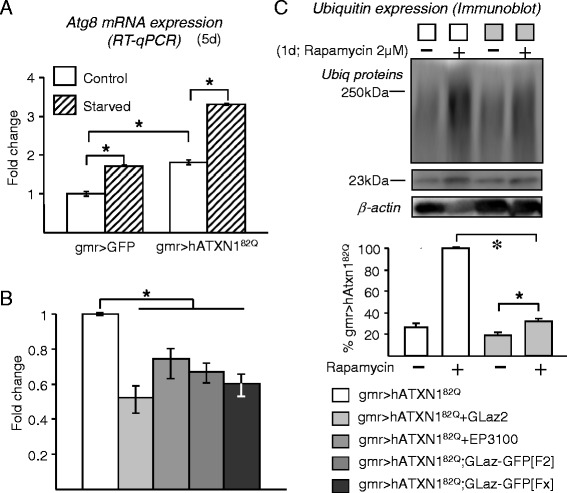
**Effects of GLaz over-expression on Atg8a expression and under pharmacological induction of autophagy. A**, Pathogenic expression of hATXN1^82Q^ and starvation increase autophagic activity, monitored by Atg8a mRNA levels. **B**, Atg8a expression is decreased by co-expression of GLaz and the hATXN1^82Q^ transgene. Statistical differences were assayed by Mann–Whitney U-test. *P < 0.05. **C**, Levels of ubiquitinated proteins in fly heads, measured by immunoblot after treatment with the autophagy inducer rapamycin. An increase in ubiquitin load is observed when SCA1 model flies are exposed to rapamycin, while a decrease in ubiquitin signal is clear upon GLaz over-expression. Color code applies for blot and graphs in B and C. Statistical differences were assayed by Student’s t-test. *P < 0.05.

In order to understand the protective mechanism of Lipocalins we studied the endogenous expression of the Drosophila Atg8a gene, a homologue of LC3 in vertebrates [38], as a first approximation to the levels of autophagy activity. Transcription of Atg8a is known to be strongly up-regulated upon starvation [39] and to show a strong correlation with autophagy activity. Although autophagy initiation might not be dependent on Atg8a up-regulation, this transcriptional response contributes to replenish the protein consumed by autophagic activity [5]. By measuring the transcription levels of the Atg8a gene we can therefore assess whether autophagic activity is increased in the SCA1 model of photoreceptor degeneration. A twofold Atg8a induction is observed (Figure 6A). This indication of increased autophagic activity is in agreement with the observed induction of GstS1 (Figure 4C), which upon oxidation would contribute to JNK-based autophagy induction, and supports the view that inducing autophagy might be a general response in poly-Q based pathologies of different etiologies [3]. Neurodegeneration-triggered Atg8a induction in the fly head is similar in extent to the induction obtained upon starvation in this tissue (Figure 6A), although quantitatively smaller than the changes observed in larval and fat body tissues [39]. If flies expressing hATXN1^82Q^ are starved, Atg8a transcription is further increased, suggesting that different mechanisms of autophagy induction are acting in an additive manner.

When GLaz is over-expressed in the photoreceptors or in retinal support cells and basal glial cells, the SCA1-dependent Atg8a induction is significantly reduced (Figure 6B). Thus, a paradox arises from the data presented so far (Figures 5 and 6), since GLaz gain-of-function reduces accumulation of ubiquitinated proteins, but at the same time reduces the levels of an indicator of autophagic activity induction (Atg8a) and an oxidative stress defensive enzyme that affects autophagic activity (GstS1). When autophagy is further stimulated by feeding SCA1 model flies with rapamycin (a TOR signalling inhibitor; see [40] for a review), two important consequences are observed: 1) more autophagic induction results in higher levels of ubiquitinated proteins, instead of enhanced clearance, and 2) GLaz over-expression reduces the accumulation of ubiquitinated proteins (Figure 6C). The first observation suggests that autophagy promotion alone is not beneficial in the SCA1 neurodegeneration model, leading instead to autophagic stress [6,7] that ultimately makes ubiquitinated proteins clearance less efficient. Although rapamycin treatment, in addition to autophagy induction, might have wider effects on protein homeostasis, it is clear that the combination of rapamycin treatment with GLaz over-expression results in an enhanced clearance of ubiquitinated proteins (Figure 6C).

These data could support different scenarios: (i) The GLaz antioxidant properties contribute to maintain induction of autophagy and other antioxidant systems at an appropriate level where these mechanisms are most effective. (ii) GLaz is not directly acting on the induction or initiation steps of autophagy, but rather promoting its resolution, making the process to progress more efficiently. (iii) The GLaz contribution to the protein quality control systems is independent of autophagy (e.g., by potentiating proteasomal function).

### GLaz prevents p62 accumulation upon SCA1-dependent and rapamycin-stimulated autophagy

**Figure 7:**
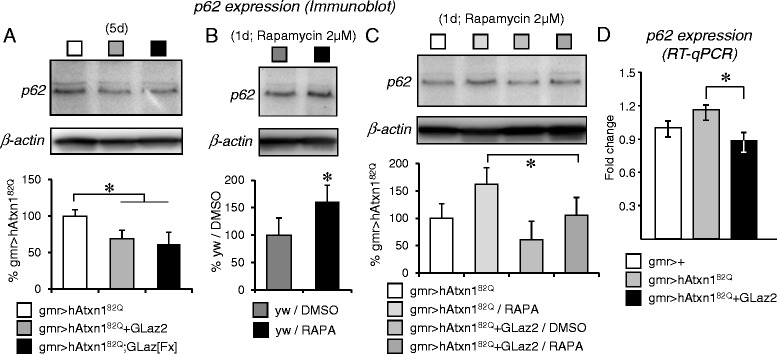
**GLaz over-expression decreases p62 protein accumulation in response to ATXN1 degeneration and autophagy induction, and modifies p62 mRNA levels in neurodegenerative conditions. A**, p62 accumulation, measured by immunoblot, decreases in SCA1-affected retinas that over-express GLaz. **B**, p62 protein expression increases upon rapamycin treatment in wild type flies. **C**, Treatment of SCA1 flies with rapamycin, combined with GLaz over-expression, further reduces p62 accumulation. **D**, A decrease in p62 mRNA expression is observed in SCA1 flies that over-express GLaz. Statistical differences were assayed by Student’s t-test (immunoblots) and Mann–Whitney U-test (RT-qPCR). *P < 0.05.

To discern between the alternatives stated above we monitored the accumulation of p62, a protein acting as a decision point for the degradation of aggregasomes (aggregates of misfolded proteins too large to be degraded by the proteasome system). p62 docks aggregasomes to newly formed autophagic membranes, thus controlling their entrance in the autophagosome [41-43]. If autophagy is working properly, p62 should also be degraded in the process. Therefore, measurement of endogenous p62 can be used to quantitatively assess the flow of autophagy of protein aggregates [44,45] serving as a readout of autophagic degradation efficiency [5]. The hypothesis that GLaz might be promoting the resolution of autophagy predicts a reduction in p62 accumulation. Levels of p62 show a 30-40% decrease upon GLaz over-expression (Figure 7A). Moreover, p62 protein levels increase upon rapamycin-induced autophagy in both, wild type control flies (Figure 7B) and in flies undergoing photoreceptor neurodegeneration (Figure 7C), as expected by the known transcriptional up-regulation of p62 in autophagy-inducing conditions [39]. Over-expression of GLaz reduces p62 levels in the SCA1 model with or without further induction of autophagy by rapamycin (Figure 7A,C). The effect of GLaz over-expression on autophagy markers does not support its role in clearance of aggregate-prone proteins by an autophagy-independent proteasome mechanism.

However, we need to discern whether p62 protein level changes are accompanied by transcriptional changes upon GLaz over-expression. We then measured the p62 transcript levels by qRT-PCR (Figure 7D) and found no significant changes upon hATXN1^82Q^ or GLaz over-expression alone, while a 25% decrease is observed by over-expressing GLaz in the SCA1 model compared to the neurodegenerative condition. These results show that GLaz over-expression results in both p62 mRNA and protein levels decrease in the SCA1 model.

### In the absence of neurodegeneration, a loss of GLaz function increases Atg8a mRNA and p62 protein levels

**Figure 8:**
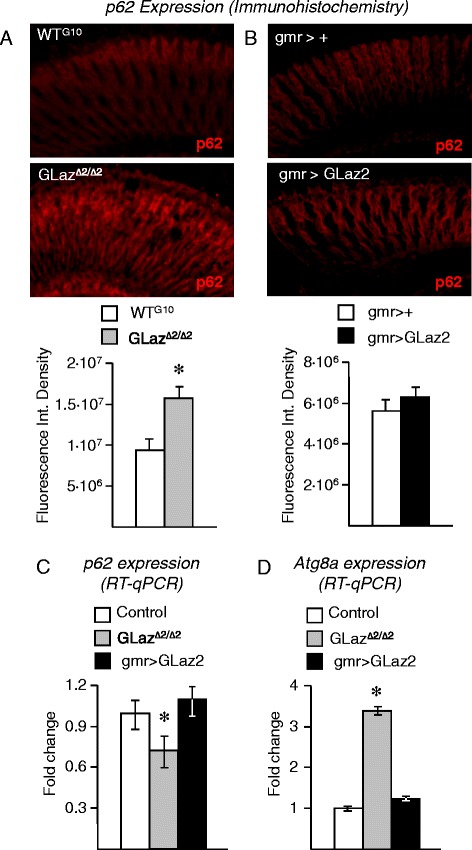
**Effects on Atg8a and p62 expression of GLaz loss- and gain-of-function in control conditions. A-B**, Immunofluorescence of control (either WT^G10^ or gmr > +), GLaz loss-of-function (GLaz^Δ2/Δ2^) and GLaz gain-of-function (gmr > GLaz2) retinas showing p62 protein expression. **C**, mRNA levels of p62, measured by RT-qPCR in control and GLaz loss- and gain-of-function fly heads. **D**, mRNA levels of Atg8a, measured by RT-qPCR in control, GLaz loss- and gain-of-function fly heads. Statistical differences were assayed by Student’s t-test (immunofluorescence) and Mann–Whitney U-test (RT-qPCR). *P < 0.05.

In basal conditions, when no neurodegeneration-triggered autophagy activity is present, GLaz null mutant flies show higher levels of p62 protein (Figure 8A), while over-expressing GLaz under basal conditions does not change p62 protein levels (Figure 8B). Transcript levels of p62 slightly decrease in GLaz null mutants, but do not change significantly by GLaz gain-of-function in the absence of neurodegeneration (Figure 8C). Atg8a transcript levels increase in GLaz null mutant flies, but no changes in Atg8a mRNA levels are observed in flies over-expressing GLaz (Figure 8D). These results suggest, on the one hand, the existence of a GLaz-dependent effect on basal autophagy progression, since the absence of GLaz expression results in increased Atg8a expression and accumulation of p62 without a parallel increase in p62 transcription. On the other hand, increasing GLaz expression levels does not affect, by itself, the expression of genes monitoring autophagic activity or the accumulation of p62 protein in the absence of a deteriorating cellular context.

### Knocking-down key autophagy genes rescues SCA1-triggered neurodegeneration, and further GLaz-dependent rescue is abolished

**Figure 9:**
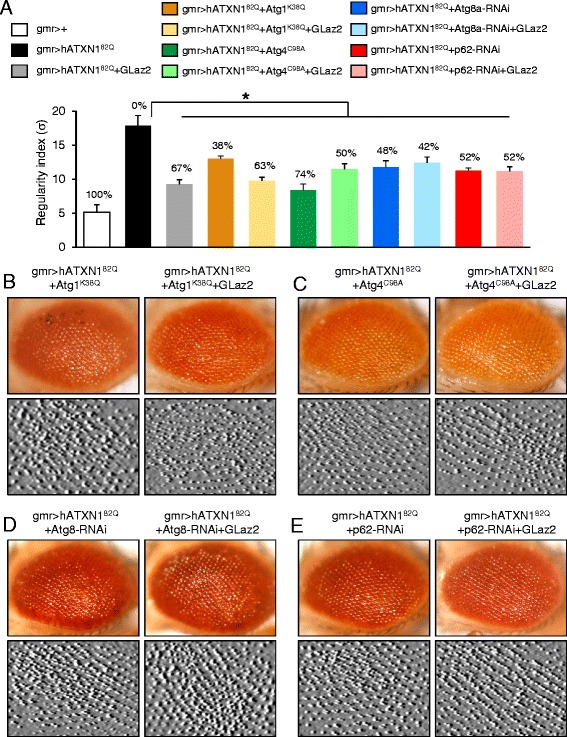
**Epistasis analysis of GLaz with down-regulators of autophagy. A**, Quantitative estimate of degeneration by computing a regularity index based on the variance (σ) of the local intensity maxima distances. A percent recovery of the degeneration is indicated for each genotype. 20–35 eyes/genotype were used to compute regularity indexes. Average ± S.E.M. are represented. Asterisk represents statistically significant differences (Student’s t-test, *P < 0.05) with respect to the degenerated gmr > hATXN1^82Q^ genotype. **B-E**, Adult (3d old) Drosophila eye morphology of gmr > hATXN1^82Q^ + autophagy down-regulators, compared to gmr > hATXN1^82Q^ + GLaz2 + autophagy down-regulators. Representative examples of light microscopy and surface filter-processed image of the central retinal region are shown.

We used four genetic manipulations to decrease autophagy activity in our SCA1 Drosophila model: Atg1^K38Q^, a kinase-negative form of Atg1 that inhibits starvation- and rapamycin-induced autophagy [46] by impairing the induction of autophagosome formation; Atg4a^C98A^, a dominant-negative form of Atg4a suppressing autophagy by defective processing of Atg8a/LC3 [47]; RNAi silencing of Atg8a (JF02895 line) [Dr. Juhász personal communication] and of p62 (KK105338 line) [47], that reduce Atg8a or p62 up-regulation respectively and impair the replenishment of both proteins when they are consumed by autophagic activity. The four constructs were expressed with the UAS/GAL4 system in the neurodegenerating photoreceptors with or without over-expression of GLaz (Figure 9).

All forms of autophagy reduction tested produce a significant rescue of photoreceptor degeneration (Figure 9), suggesting that in this neurodegenerative condition, autophagy must be contributing to neuronal death. These data, together with the observed accumulation of ubiquitinated proteins upon autophagy induction in the SCA1 model flies, suggest that hATXN1^82Q^ expressing cells are suffering autophagic stress [6,7]. GLaz over-expression under all four conditions of autophagy down-regulation results in a rescue of the rough eye phenotype, that is either similar or even slightly better than when autophagy is not genetically modified. No significant additive effect of both rescuing manipulations is observed, thus reflecting epistatic relationship between GLaz and autophagy control genes.

## Discussion

We have used the Drosophila retina as a cellular substrate to model the poly-Q-based SCA1 neurodegenerative disease, and found that two lipid-binding proteins, the Drosophila Lipocalins GLaz and NLaz, are able to counteract cellular damage and maintain cell viability and tissue integrity to a reasonable extent. Both Lipocalin genes are part of the endogenous transcriptional response to the neurodegenerative insult triggered by the expanded poly-Q human Ataxin 1. The beneficial effects of GLaz occur when the Lipocalin is either expressed by the degenerating neuron, or in a paracrine manner by the nearby support cells or basal glial cells. Neurodegeneration rescue is abolished by a GLaz interfering RNA construct, further supporting the effect specificity. Moreover, the neuroprotective effect of GLaz is persistent as the animal ages.

The fact that nuclear inclusions of poly-Q-Ataxin 1 are still present in GLaz over-expressing flies, suggests a role for GLaz in processes occurring in the cytoplasm of the affected cells. We have detected co-localization of the GLaz-GFP fusion protein with photoreceptor markers in the neurodegenerative SCA1 model. Likewise, human ApoD is internalized by murine astrocytes, and the internalized exogenous Lipocalin is observed in intracellular membranous and vesicular compartments, but not inside the nucleus [18]. A similar intracellular traffic of GLaz is expected, and therefore worth to study. Interestingly, we have also recently found that ApoD, in addition to perform extracellular functions in injured peripheral nerves (controlling lipid mediators), promotes myelin degradation by macrophages [22], an intracellular process that also involves the formation of phagosomes.

Induction of autophagy has been shown in a Huntington disease model in response to the pathogenic mutant version of huntingtin [3], and induction of mRNA and protein p62 expression have been demonstrated upon expression of poly-Q expanded huntingtin [48]. On the other hand, experimental induction of autophagy is beneficial in most, but not all, proteinopathies tested so far [4]. Until now, the role of autophagy in SCA1 pathogenesis was unclear. Here we show that autophagy is also part of the tissue response to the expression of the human pathogenic version of Ataxin 1. We demonstrate that inhibition of the autophagy process *in vivo* rescues the fly retina from SCA1-triggered damage and that induction of autophagy in SCA1 flies compromises ubiquitinated proteins clearance. These results agree with the conclusions reached by recent works showing that activation of autophagy in neurons under autophagic stress compromises neuronal survival [49]. Similarly, autophagy induction, autophagosome accumulation and increased levels of ubiquitinated proteins are accompanied by decreased mTOR signaling [50]. Therefore, our data support that cells might be undergoing autophagic stress in this model [6,7], and that this cellular response is probably lowering the threshold for the onset of apoptotic cell death [7]. This information is relevant in the context of the use of this Drosophila retinal degeneration model of SCA1 to search for genetic modifiers [30,33].

Our data support the view that GLaz beneficial effect on SCA1 neurodegeneration concurs with the modulation of neurodegeneration-triggered selective autophagy. GLaz shows epistatic relationships with autophagy genes involved in the induction of phagofore formation; Atg8a/ LC3 processing (conditioning the expansion into autophagosomes); targeting aggregated proteins cargo into the phagofore; and replenishing Atg8 and p62 levels upon autophagic activity. Moreover, in the SCA1 model, over-expressing GLaz lowers endogenous Atg8a and p62 transcript levels, as well as p62 protein levels, suggesting a decrease in autophagic activity that might counteract excessive autophagy induction. The loss of GLaz function increases Atg8a mRNA levels and leads to p62 protein accumulation in basal conditions, also suggesting a role in the modulation of basal autophagic activity. Although the observed decrease in p62 protein upon GLaz over-expression in SCA1 model flies, or the p62 protein accumulation in GLaz null mutants in basal conditions, could also be interpreted as signs of autophagy flux alterations [5], the small but parallel changes in p62 transcription under neurodegenerative conditions cast doubts on this scenario as the sole explanation.

We have previously shown that GLaz and NLaz have beneficial effects under oxidative stress elicited either experimentally, through normal aging [12-16], or evoked by Friedreich Ataxia, a mitochondrial dysfunction-based neurodegenerative disease [29]. The control of lipid peroxidation levels lies at the base of these outcomes. Here we find that the Lipocalin GLaz is also able to rescue photoreceptors from pathogenic SCA1-induced apoptotic cell death by an apparently different mechanism. Interestingly, several control points of autophagy are either directly regulated by the cell redox state or are part of feedback regulatory loops in which oxidative stress or lipid peroxide levels are involved: (i) The activity of the cysteine protease Atg4a is redox sensitive [51,52]. (ii) The expression of p62 is induced by oxidative stress [53] and, in turn, p62 works as a signaling molecule promoting antioxidant response through its effect on Nrf2 transcription factor activation [54]. (iii) GstS1 activity modulates autophagy though its regulation of the JNK pathway [34]. Interestingly, GLaz null mutants display increased oxidative stress sensitivity, higher levels of lipid peroxidation and apoptotic cell death [12]; phenotypes that are often associated with autophagy malfunction. Thus, a parsimonious hypothesis would be that the Lipocalin-mediated control of lipid peroxide levels influences autophagy at several steps, slowing down the process and ultimately making it more efficient. These functions will promote clearance of aggregated proteins and would prevent crossing the threshold to apoptotic cell death programs. We thus propose that GLaz participates in the optimization of autophagy, contributing to make this process efficient and preventing autophagic stress, which would, in due time, develop alongside the severe degeneration produced by a relentless hATXN1^82Q^ expression under the UAS-GAL4 system.

It is known that autophagy defects lead to neurodegeneration even in cases where poly-Q protein expansions are not involved; e.g., in the Pink1 and Parkin loss-of-function leading to Parkinson disease, where mitophagy defects are proposed to induce neuronal death [55,56]. Therefore, the Lazarillo-related Lipocalins, with their potential to modulate autophagy, might be useful in many other neurodegenerative diseases. Also, it is known that autophagy efficacy decreases with aging [57]. How is autophagy activity modulation contributing to the well-known longevity modulation phenotypes of GLaz and other Lazarillo-related Lipocalins [12-16] becomes an interesting future research avenue. Another member of the Lipocalin family, Lcn2, has been involved in autophagy modulation in murine embryonic cells, where loss of Lcn2 function results in a decrease in LC3 protein expression [58].

In summary, the Lazarillo-related Lipocalins, known to protect membranes from lipid peroxidation and its collateral damages, are also contributing to prevent autophagic stress under poly-Q-based neurodegenerative conditions. Understanding this pleiotropic nature of ApoD and Lazarillo-related Lipocalins will help us to better explain why ApoD is a key element in the nervous system response to such a wide array of neurodegenerative and injury-triggered diseases.

## Conclusions

From our analysis of Lazarillo-related Lipocalins in the Drosophila retina model of SCA1 we can conclude that:

1. Two extracellular lipid binding proteins, GLaz and NLaz, are able to counteract cellular damage and maintain tissue integrity when the retina is suffering from the deleterious effects of expressing a poly-Q-expanded version of human Ataxin 1.
2. Both Lipocalins are part of the endogenous transcriptional response to the neurodegenerative insult, as ApoD is in many other nervous system diseases. GstS1, which contributes to lipid-peroxide clearance, is also induced in the SCA1 model.
3. The beneficial effects of GLaz can be exerted both, from within the degenerating neuron, or in a paracrine manner from the nearby support and basal glial cells.
4. The neuroprotective effect of GLaz is persistent with age.
5. GLaz gain-of-function reduces the amount of ubiquitinated proteins in neurodegenerative conditions.
6. GLaz loss-of-function increases Atg8a mRNA, and leads to p62 protein accumulation without a concomitant increase in its mRNA levels.
7. GLaz gain-of-function decreases SCA1-dependent induction of Atg8a and p62 mRNA and decreases p62 protein levels in the degenerated tissue.
8. Genetic down-regulation of autophagy rescues SCA1-triggered neurodegeneration, and pharmacological up-regulation of autophagy does not promote ubiquitinated proteins clearance in the SCA1 model, suggesting that cells are undergoing autophagic stress.
9. Epistasis genetic analysis with down-regulators of the autophagy process demonstrates that GLaz beneficial effects concur with a restrain in the extent of selective autophagy activity, which ultimately leads to an improved clearance of aggregation-prone proteins.
10. GLaz is able to improve ubiquitinated protein clearance even when combining SCA1-dependent with pharmacological inductions of autophagy. This result opens the possibility of an effective combination of therapeutic interventions, not only for SCA1, but for many other neurodegenerative diseases.
11. Lazarillo-related Lipocalins, including ApoD, can now be understood as useful and efficient players in the endogenous protection against the two major factors contributing to aging and neurodegeneration: ROS-dependent damage and proteostasis deterioration.

## Methods

### Fly strains and husbandry

Flies were grown under standard laboratory conditions as described [12]. Fly females were used in all experiments. Unless stated otherwise, experiments were performed at 25°C.

The null mutant GLaz^Δ2/Δ2^ and its isogenic wild type control (WT^G10^) in Canton S background were previously described [12]. We used a construct (glaz:GLaz-GFP) containing 1.9 kb of the native 5’ genomic region upstream and the full-length GLaz gene, in frame with the coding sequence of GFP, thus producing a GLaz-GFP fusion protein expressed under the control of the native GLaz regulatory sequences [12]. Two independent transformant lines with insertions of this construct were used: 1) glaz:GLaz-GFP[FX] and 2) glaz:GLaz-GFP[F2], located in the first and second chromosome respectively.

NLaz expression was characterized by various reporter constructs, with 3 kb of the native 5’ genomic region upstream of NLaz followed by GFP (nlaz:GFP[R2]). Alternatively, we used the same NLaz 5’ genomic region to drive the expression of GAL4 to express a membrane bound GFP (UAS:CD8-GFP line).

We used the line gmr:GAL4 to drive transgenes expression to the eye photoreceptors. The UAS:GLaz and UAS:NLaz lines have been reported [13,16]. The UAS:hATXN1^82Q^; gmr:GAL4 line was kindly provided by Dr. J. Botas. UAS:Atg1^K38Q^ [46], UAS:Atg4a^C98A^ [47], UAS:Atg8a RNAi^JF02895^, and UAS:p62 RNAi^KK105366^ [47,59] were kindly provided by Dr. Gábor Juhász. All other fly lines were obtained from the Bloomington stock Center.

To combine the neurodegeneration model with GLaz over-expression and with different forms of autophagy inhibition (or other control transgenes) we constructed a fly line UAS:hATXN1^82Q^; gmr:GAL4 UAS:GLaz2/CyO (with two transgenes in the second chromosome). After the scheme of crosses, presence of GLaz cDNA transgene was confirmed by PCR amplification from genomic DNA.

### Immunohistochemistry

Fly heads were fixed with 4% paraformaldehyde and embedded in paraffin following standard procedures. Tissue sections (4 μm) were performed with a rotary microtome (Microm), serially mounted on Polysine™ slides (Menzel-Gläser), and dried. The sections were dewaxed in xylene and rehydrated through an ethanol series into phosphate buffered saline (PBS). Larval optic imaginal discs were dissected and fixed in 4% paraformaldehyde. The tissue was then blocked and permeabilized with TritonX-100 (0.1% in PBS) and 2% normal goat serum.

The following primary antibodies were used: Rabbit serum anti-p62 (a kind gift of Dr. Juhasz, Loránd University, Hungary; [47]); Rabbit serum anti-Atxn1 (a kind gift of Dr. J. Botas, Baylor College of Medicine, USA); Mouse monoclonal P4D1 anti-Ubiquitin (Cell Signaling); Rabbit serum anti-GFP (Santa Cruz Biotechnology, Inc.); Mouse monoclonal 4C5 anti-Rhodopsin (DSHB); Mouse monoclonal 22C10 anti-MAP1B (DSHB).

For HRP-IHC, the secondary antibodies HRP-conjugated Goat anti-Rabbit IgG (Abcam) and HRP-conjugated Goat anti-Mouse IgG (Dako) were used. HRP development was achieved with DAB (0.03%) and H_2_O_2_ (0.002% in 50 mM Tris, pH 8.0). Hematoxilin/Eosin and HRP-IHC sections were mounted after dehydration and clearing in Eukitt™.

For fluorescence IHC, the following secondary antibodies were used: FITC-conjugated Goat anti-Mouse and Cy3.5-conjugated Goat anti-Rabbit (Abcam). After washes in PBS, the sections were mounted with Vectashield-DAPI (Vector Labs).

The TUNEL FITC labeling kit (Roche) was used to assay apoptotic cell death in fly head tissue sections according to the manufacturer’s protocol.

Labeled sections were visualized and photographed with an Eclipse 90i (Nikon) fluorescence microscope equipped with DS-Ri1 (Nikon) digital camera. Images were acquired and processed with the NIS-Elements BR 3.0 software (Nikon).

### Eye external morphology

Flies were anesthetized with CO_2_ and immobilized with adhesive tape. Fly eyes were photographed with a Nikon DS-L1 digital camera, in a Nikon SMZ1000 stereomicroscope equipped with a Plan Apo 1× WD70 objective. Images were processed with Adobe Photoshop 6.0 using a surface filter. Local intensity maxima were obtained with ImageJ software, and nearest neighbor distances were calculated for each ommatidia. A regularity index was estimated as the variance of distances, and a percent recovery was calculated considering 0% the average degenerated eye and 100% the control wild type eye. Samples of 20–35 flies (3d old) per condition and genotype were used to calculate the average ± S.E.M. regularity index. We have developed an optimized version of this methodology, with a freely available ImageJ plugin for automated analysis of fly eye pictures [31].

For scanning electron microscopy images, the flies were immobilized and pictures were taken with an ESEM-FEI-Quanta 200FEG scanning electron microscope.

### Quantitative RT-PCR

Drosophila heads (30–50 per condition and genotype) used for mRNA expression studies were stored at −80°C, and RNA was extracted using QIAzol™ Lysis Reagent (Qiagen). RNA concentration was measured with a Nanodrop spectrophotometer and its integrity assayed by gel electrophoresis. Following DNAse treatment, 500 ng of total RNA was reverse-transcribed with PrimeScript™ (Takara Bio Inc., Otsu, Japan) according to the manufacturer’s instructions using Oligo-dT primers and random hexamers. The resulting cDNA was used as template for RT-qPCR using SybrGreen (SYBR® Premix Ex Taq™ kit, Takara) in quintuplicate PCR reactions in a Rotor-Gene RG-3000 thermal cycler (Corbett Research). Cycling conditions were 30 sec 95°, (5 sec 95°, 15 sec 55°, 15 sec 72°) × 40. Melting curves were established for all conditions to check for the absence of unspecific amplifications. The primers used for RT-qPCR are shown in Table 1. The gene Rpl18 was used as the reference gene.

**Table 1:** **RT-qPCR primer sequences**

**Gene**	**Primer orientation**	**Primer sequence**
NLaz	Forward	5′-CGAGTACGCAGCCTATCCAT-3′
	Reverse	5′-CCAGGTAGTTGGCCTTCGT-3′
GLaz	Forward	5′-GCGAACAATCGAAGTTTTCC-3′
	Reverse	5′-ACAAGATGGCGAAGTTCTCG-3′
hATXN1	Forward	5′-GTGGCCGTGATACAGTTCG-3′
	Reverse	5′-AGCCGTTCTTCAGGTTCTTG-3′
GstS1	Forward	5′-AAGGACAACGATGGTCACCTGGC-3′
	Reverse	5′-CGGTGAACTTAGACCTCGGTGACG-3′
Vib	Forward	5′-GGCCAATCGCACTCCCCAGTTC-3′
	Reverse	5′-TAACACCTCGATGCCCTCGCC-3′
Atg8a	Forward	5′-CATCGGTGATTTGGACAAGA-3′
	Reverse	5′-AGTCCTCCTCGTGATGTTCC-3′
p62	Forward	5′-CGTAAGGACCTTCTGGATCG-3′
	Reverse	5′-CGTCGTGGATGGTGAAATTG-3′
L18	Forward	5′-AGAACCGAGCCCAAATCC-3′
	Reverse	5′-CGACCACGATGGTAGACTCC-3′

Transcription levels of mRNA were calculated with the ΔΔC_T_ method [60]. Statistically significant differences of gene transcriptional changes were evaluated with a Mann–Whitney U-test [61], using ΔC_T_ of each replica (calculated by subtracting the average CT of the reference gene). The statistical level of significance was set at *P* < 0.05.

### Immunoblot analysis

Proteins from fly heads (30–50 per condition and genotype) were solubilized by homogenization in lysis buffer, and total protein concentration was evaluated by BCA analysis (Pierce).

Immunoblot analyses were performed with 10–20 μg of total protein/lane transferred to PVDF membranes using standard procedures and exposed to the following antibodies: Rabbit serum anti-p62 (Dr. Juhasz, Loránd University, Hungary; [47]); Mouse monoclonal P4D1 anti-Ubiquitin (Cell Signaling). HRP-conjugated Goat anti-Rabbit or mouse IgG (DAKO) were used as secondary antibodies. HRP-conjugated anti-β actin antibody (Sigma, St Louis, MO, USA) was used to normalize protein loads. Membranes were developed with ECL (Millipore, Billerica, MA, USA), and the signal visualized with a digital camera (VersaDoc, BioRad). The integrated optical density of the immunoreactive protein bands was measured in images taken within the linear range of the camera to avoid signal saturation.

### Statistical analysis

Statistical tests were performed with SigmaPlot (v 11.0) software. A *P-value* < 0.05 was defined as a threshold for significant changes. Means and dispersion values were calculated from experimental triplicates. The particular tests used are stated in figure legends.

## Additional files

Additional file 1:
**GLaz expression does not alter early levels of human Ataxin 1 expressed in photoreceptors by the GAL4/UAS system.** A-C, Third instar eye imaginal discs labeled with anti-hATXN1 polyclonal antibody 11NQ. hATXN1 is not detected in driver-only controls. Photoreceptors are immunopositive when hATXN1^82Q^ is expressed by the gmr driver. A similar fluorescence level is observed when both hATXN1^82Q^ and GLaz2 transgenes are co-expressed. D, Relative amounts of immunofluorescence signal quantified in a 400 μm^2^ area/disc. Statistical differences assayed by Student’s t-test. **P* < 0.05. Additional file 2:
**Effect of different forms of GLaz and NLaz gain-of-function in hATXN1** ^**82Q**^ **-driven photoreceptor degeneration.** Representative examples of Drosophila adult eyes of different genotypes. Light microscopy (upper panels) and surface image (lower panels) are shown in all cases. A, SCA1-affected retinas and lack of rescue upon LacZ over-expression. B, NLaz gain-of-function rescue with two different trangene lines. C, GLaz gain-of-function rescue with a GLaz trangene and two EP insertion lines. D, GLaz gain-of-function rescue when two copies of SCA1 transgene are present. Additional file 3:
**GLaz loss-of-function does not rescue the hATXN1** ^**82Q**^ **photoreceptor degeneration and GLaz RNAi counteracts rescue of GLaz over-expression.** Representative examples of Drosophila adult eyes. A, Light microscopy (upper panels) and surface image (lower panels) are shown in GLaz loss-of-function control retinas (gmr driver) and upon expression of pathogenic hATXN1^82Q^ in GLaz null-mutant background. B, GLaz gain-of-function rescue of neurodegeneration is reverted when GLaz RNAi is co-expressed in the fly retina. Additional file 4:
**Expressing GLaz with its native spatiotemporal pattern rescues hATXN1** ^**82Q**^ **-dependent photoreceptor degeneration throughout aging.** A-D, Examples of Drosophila eyes of degenerated (gmr > hATXN1^82Q^) and two different GLaz expression transgenes (glaz:GLaz-GFP[FX] and glaz:GLaz-GFP[F2]) of pupa (5 dAPF) (A), 1 day (B), 3 days (C) and 30 days (D) old flies.

## Abbreviations

APF: Days after pupa formation
ApoD: Apolipoprotein D
Atg8a/LC3: Autophagy-specific gene 8a/ Microtubule-associated protein 1A/1B-light chain 3
CNS: Central nervous system
DRPLA: Dentatorubral-pallidoluysian atrophy
GAL4: *Saccharomyces cerevisiae* transcriptional regulatory protein
GFP: Green Fluorescent Protein
GLaz: Glial Lazarillo
gmr: Glass multimer reporter promoter
GstS1: Glutathione S transferase S1
hATXN1^82Q^: Human Ataxin 1 with 82 glutamine tract
HPGDS: mammalian hematopoietic prostaglandin D synthase
JNK: Jun-N-terminal Kinase
LacZ: Beta-D-galactosidase from *Escherichia coli*
Lcn1: Lipocalin 2
NLaz: Neural Lazarillo
Pink1: PTEN induced putative kinase 1
PNS: Peripheral nervous system
poly-Q: Polyglutamine
ROS: Reactive oxygen species
SCA1: Type 1 Spinocerebellar Ataxia
TOR: Target of rapamycin
TUNEL: Terminal deoxynucleotidyl transferase dUTP nick-end labeling
UAS: Upstream activating sequences
Vib: Vibrator
