# Supplementary figures and images for: Lazarillo-related Lipocalins confer long-term protection against type I Spinocerebellar Ataxia degeneration contributing to optimize selective autophagy
Source: Mol Neurodegener. 2015 Mar 19;10:11. doi: 10.1186/s13024-015-0009-8 (PMC4374295; doi:10.1186/s13024-015-0009-8)

Figure S1

*hATXN1* immunolabeling (optic discs, L3 larva)

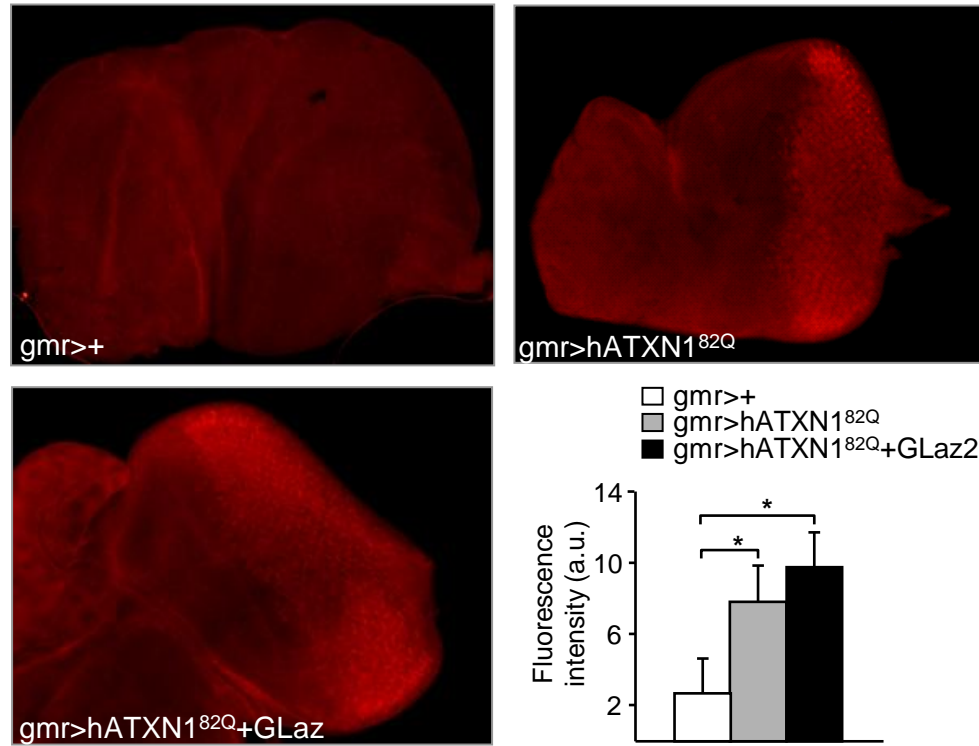

Supplement: Additional file 1: — GLaz expression does not alter early levels of human Ataxin 1 expressed in photoreceptors by the GAL4/UAS system. A-C, Third instar eye imaginal discs labeled with anti-hATXN1 polyclonal antibody 11NQ. hATXN1 is not detected in driver-only controls. Photoreceptors are immunopositive when hATXN182Q is expressed by the gmr driver. A similar fluorescence level is observed when both hATXN182Q and GLaz2 transgenes are co-expressed. D, Relative amounts of immunofluorescence signal quantified in a 400 μm2 area/disc. Statistical differences assayed by Student’s t-test. *P < 0.05. [file 13024_2015_9_MOESM1_ESM.pdf]

Figure S2

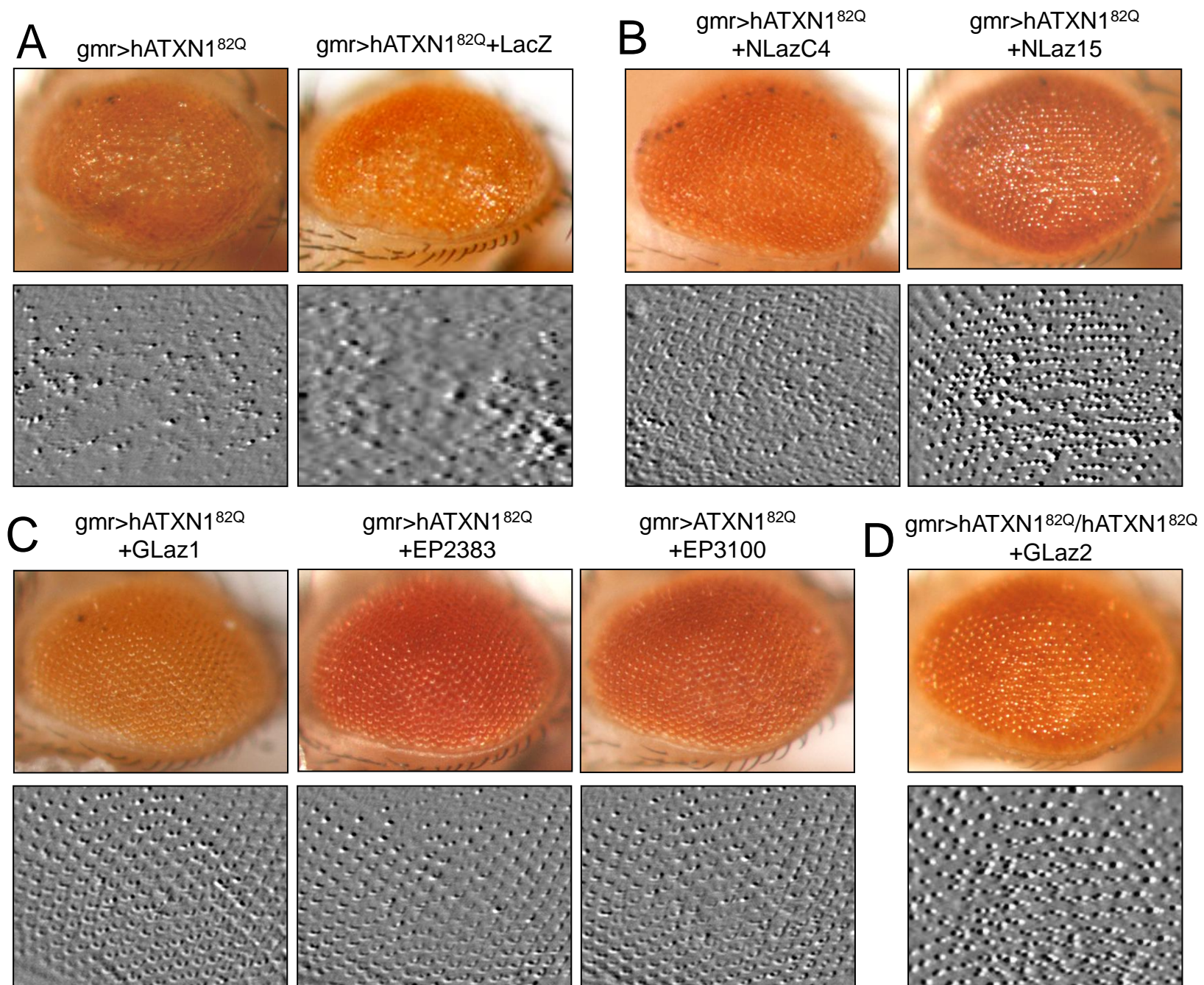

Supplement: Additional file 2: — Effect of different forms of GLaz and NLaz gain-of-function in hATXN1 82Q -driven photoreceptor degeneration. Representative examples of Drosophila adult eyes of different genotypes. Light microscopy (upper panels) and surface image (lower panels) are shown in all cases. A, SCA1-affected retinas and lack of rescue upon LacZ over-expression. B, NLaz gain-of-function rescue with two different trangene lines. C, GLaz gain-of-function rescue with a GLaz trangene and two EP insertion lines. D, GLaz gain-of-function rescue when two copies of SCA1 transgene are present. [file 13024_2015_9_MOESM2_ESM.pdf]

Figure S3

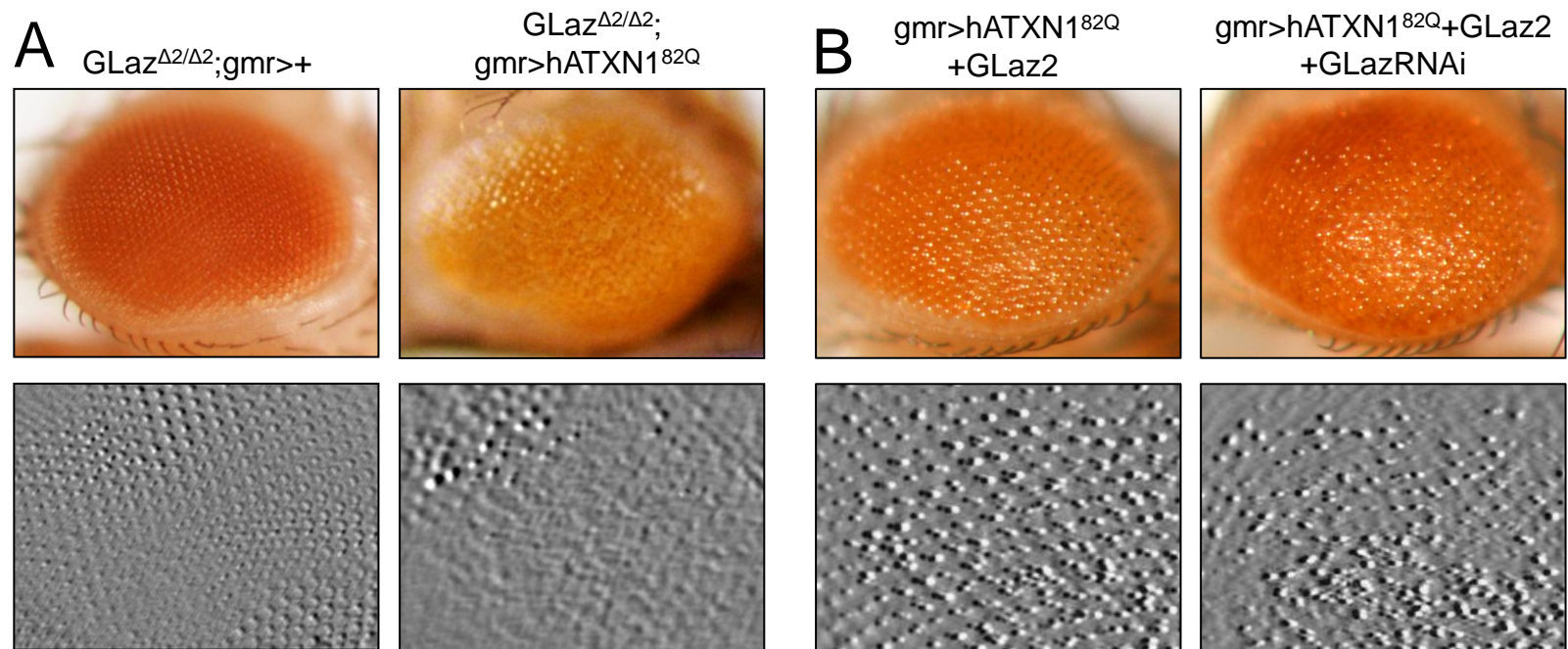

Supplement: Additional file 3: — GLaz loss-of-function does not rescue the hATXN1 82Q photoreceptor degeneration and GLaz RNAi counteracts rescue of GLaz over-expression. Representative examples of Drosophila adult eyes. A, Light microscopy (upper panels) and surface image (lower panels) are shown in GLaz loss-of-function control retinas (gmr driver) and upon expression of pathogenic hATXN182Q in GLaz null-mutant background. B, GLaz gain-of-function rescue of neurodegeneration is reverted when GLaz RNAi is co-expressed in the fly retina. [file 13024_2015_9_MOESM3_ESM.pdf]

Figure S4

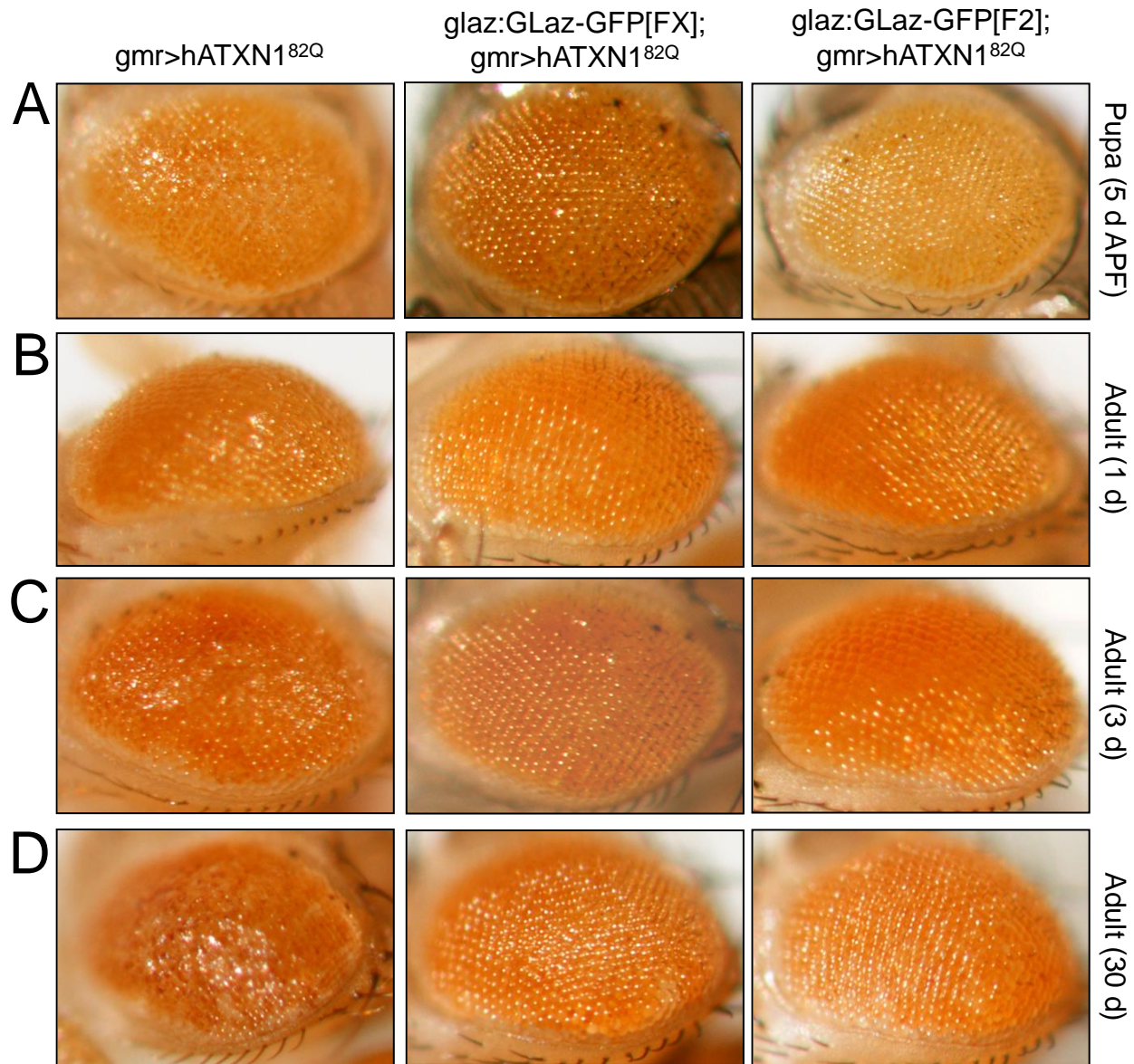

Supplement: Additional file 4: — Expressing GLaz with its native spatiotemporal pattern rescues hATXN1 82Q -dependent photoreceptor degeneration throughout aging. A-D, Examples of Drosophila eyes of degenerated (gmr > hATXN182Q) and two different GLaz expression transgenes (glaz:GLaz-GFP[FX] and glaz:GLaz-GFP[F2]) of pupa (5 dAPF) (A), 1 day (B), 3 days (C) and 30 days (D) old flies. [file 13024_2015_9_MOESM4_ESM.pdf]
